# Study of the Effect of Antibiotics in Drinking Water on the Content of Antioxidant Compounds in Red Wines

**DOI:** 10.3390/molecules28010206

**Published:** 2022-12-26

**Authors:** Marienela Calsin-Cutimbo, Nils Leander Huamán-Castilla, Jhony Mayta-Hancco, Elías Escobedo-Pacheco, Franz Zirena-Vilca

**Affiliations:** 1 Doctorado en Ingeniería de Procesos, Universidad Nacional de San Agustín de Arequipa, Av. Independencia S/N, Arequipa 04001, Peru; 2 Escuela de Ingeniería Agroindustrial, Universidad Nacional de Moquegua, Prolongación Calle Ancash S/N, Moquegua 18001, Peru; 3 Laboratorio de Contaminantes Orgánicos y Ambiente del IINDEP de la Universidad Nacional de Moquegua, Urb Ciudad Jardín-Pacocha-Ilo 18611, Peru

**Keywords:** antibiotics, polyphenols, anthocyanins, Peleg’s kinetic parameters

## Abstract

The presence of antibiotic residues in drinking water may be a source of contamination, which could affect the diffusion of polyphenols into the wine must during the traditional fermentation process. Antibiotic residues such as ivermectin, hydroxychloroquine, ciprofloxacin, and azithromycin on the diffusion of polyphenols and anthocyanins during wine fermentation were studied. Different samples were taken at different periods (0, 48, 96, and 168 h) to analyse the total polyphenols, anthocyanin content, and antioxidant capacity, which were correlated with Peleg’s equation to establish the diffusion kinetics of these compounds. The results indicated that the presence of antibiotics reduced between 40 and 50% the diffusion of the total polyphenols and monomeric anthocyanins in red wine. The use of ivermectin showed the highest kinetic parameter k_1_ compared with the use of other antibiotics. This suggested that the chemical structure and molecular weight of the antibiotics could play an important role in inhibiting the metabolism of yeasts affecting the ethanol and CO_2_ production. Consequently, cell membranes would be impermeable and would not allow the release of polyphenols and anthocyanins. Therefore, it is necessary to establish strategies that allow future water quality control in wine production companies.

## 1. Introduction

The food industry consumes between 5 and 10 m^3^ of drinking water per ton of food during the development of different unit operations and cleaning and disinfection procedures [1]. Drinking water is obtained from different sources of water that are treated through a series of stages such as precipitation, filtration, and disinfection, which are intended to guarantee its consumption [2]. However, these purification treatments only consider the microbiological content as a quality parameter, leaving aside the presence of other toxic compounds such as heavy metals, pesticides, and antibiotics [3,4,5].

Antibiotics present in drinking water are the result of their excessive use in agriculture and medicine, which can reach rivers and underground water [6]. In general, ciprofloxacin, ivermectin, and azithromycin are the compounds with the highest concentrations present in drinking water due to their use in treating infections related to bacteria and viruses [7]. For example, Boleda et al. [8] reported values of ciprofloxacin (0.1–0.34 ng/mL) and azithromycin (0.17–0.57 ng/mL) and Nippes et al. [9] reported values of ivermectin between 0.005 and 0.020 ng/mL. In addition, prolonged exposure to these compounds in the daily consumption of drinking water could represent a risk to the health of consumers [10,11,12]. Thus, during food processing, the addition of drinking water could affect the composition and quality of processed foods.

In particular, wine is a product that presents distinctive sensory characteristics of colour, aroma, and flavour as well as a particular chemical composition rich in polyphenols (1–5 g/L) [13,14]. The polyphenolic profile of wine is represented by different families of polyphenols such as anthocyanins, phenolic acids, flavonols, and stilbene, which have shown interesting bioactive properties for the treatment and prevention of degenerative diseases related to oxidative stress such as cancer, diabetes, and swellings [15,16]. However, to produce 1 litre of wine, between 2 and 6 litres of drinking water are required for the different processing stages as well as cleaning and disinfection [17].

The traditional wine process involves stages such as reception, destemming, crushing, maceration, fermentation, pressing, ageing, racking, clarification, and packaging, which define the chemical and sensory characteristics [18]. Nevertheless, traditional vineyards need to add drinking water after crushing the grapes to correct the concentration of soluble solids from 35 to 24° Brix in the must to avoid producing wine with high alcohol levels during the fermentation process [19,20]. Thus, it is possible that the presence of antibiotic residues in drinking water could alter the normal development of the fermentation process; consequently, the chemical composition of the wine would be altered.

During fermentation, yeasts partially oxidise soluble solids (sugars) to produce ethanol and CO_2_ [21,22]. This mechanism allows the release of polyphenols due to the increased permeability of cell vacuoles [23]. However, the presence of antibiotics in the water could inhibit the metabolic activity of the yeasts, reducing the production of ethanol and CO_2_; consequently, a high proportion of polyphenols would not be released into the must.

The fermentation process is based on a transfer of polyphenols from the cell membrane to the wine must. Thus, it is possible to use the Peleg model, a non-exponential empirical equation, which allows the prediction of the kinetic behaviour of polyphenol diffusion [24]. This study aimed to evaluate how the presence of antibiotic residues in drinking water affected the diffusion of polyphenols during the wine fermentation process. Thus, four antibiotics were selected as the most abundant in drinking water to analyse the diffusion kinetics of polyphenols and tentatively establish how these results could affect the permeability of the cell membrane as well as the production of ethanol and CO_2_. These results are essential to establish new strategies to control the quality of drinking water to obtain wines with a high chemical, sensory, and safety quality.

## 2. Results

### 2.1. Effect of the Concentration of Antibiotics on Total Polyphenol Content and Antioxidant Capacity

**Figure 1:**
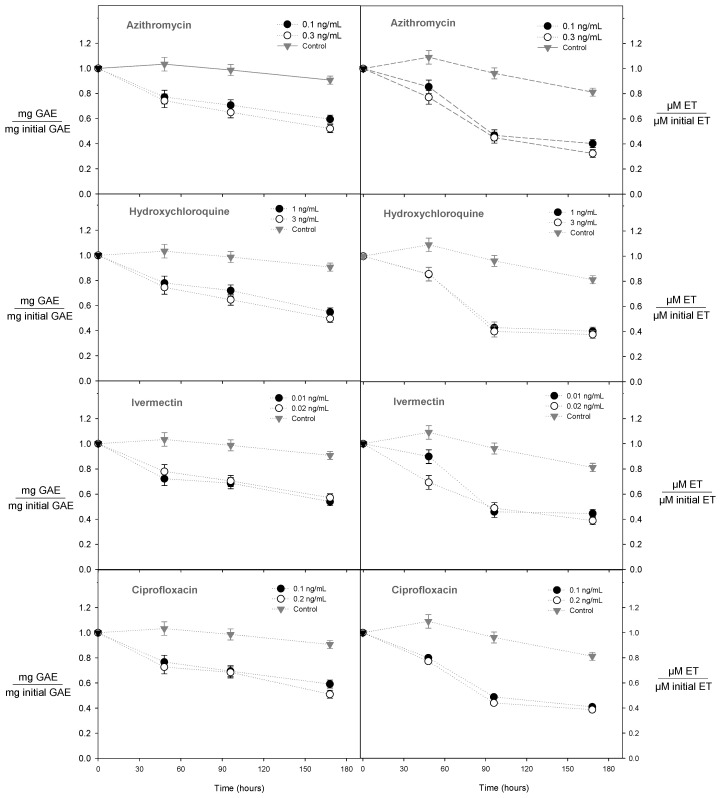
Effect of antibiotic type on total polyphenol content and antioxidant capacity during the fermentation process.

**Figure 2:**
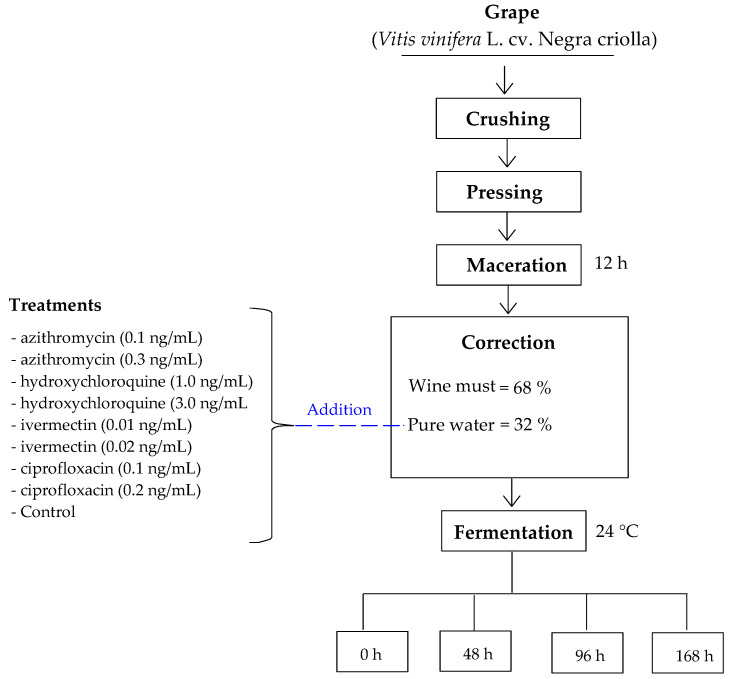
Graphical scheme of the study.

The addition of drinking water with low concentrations of antibiotics to the wine allowed us to evaluate the kinetic extraction of polyphenols during the wine fermentation process (Figure 1). According to our results, there were significant differences between the control and the different concentrations of antibiotics (*p* < 0.05). The results showed that the presence of azithromycin, hydroxychloroquine, ivermectin, and ciprofloxacin in the wine must reduced the extraction of polyphenols by 40, 45, 36, and 43% compared with the control (wine must without antibiotics), respectively (Figure 2). Interestingly, for all cases, during the first 8 h, the rate of extraction of polyphenols slowly reduced, reaching up to 15%. A rapid decline was then observed between 48 and 168 h, reaching up to 75% (Figure 1).

At the beginning of the fermentation process in the wine must and without the presence of antibiotics (control), the diffusion of polyphenols was constant due to the presence and accumulation of ethanol and CO_2_, which increased the permeability of the cell membranes, facilitating the release of polyphenols [21,23,25]. Contrarily, when antibiotics were used in the wine must, the release of polyphenols was affected. It is probable that the presence of low concentrations of antibiotics in the wine must inhibited the metabolic activity in the yeasts, causing a lower production of ethanol and CO_2_ and preventing the permeability of the cell membrane; consequently, a lower proportion of polyphenols was released into the wine must.

Although no studies have explained the inhibition mechanisms of these antibiotics on unicellular wine yeasts (*Saccharomyces cerevisiae*), there are studies that mention the effect of antibiotics on other types of unicellular yeasts associated with infections in human beings [26]. For example, Ku et al. (2010) observed in yeasts of the genus *Candida* sp. that the use of azithromycin (1000 µg/mL) could reduce its metabolic activity by ~46%; Li et al. (2015) reported that the use of hydroxychloroquine (>500 µg/mL) could inhibit the metabolic activity of these organisms by up to 80%; and Stergiopoulou et al. (2009) showed that the use of ciprofloxacin (>0.19 mg/L) could inhibit the metabolic activity of these yeasts by 50%. The primary mechanism of inhibition of these antibiotics is related to their ability to interact with ribosomal subunits; this prevents _t_RNA from interacting with ribosomes, which inhibits protein, nucleic acid, and folic acid synthesis [27,28,29]. In this sense, it is probable that the presence of low concentrations of antibiotics in the wine must inhibits the metabolic activity of yeasts, causing a lower production of ethanol and CO_2_, and preventing the permeability of the cell membrane; consequently, a lower proportion of polyphenols are released into the wine must.

Concerning the antioxidant capacity, for all the study conditions, the higher the antioxidant capacity in the wine, the higher the polyphenol content (Figure 1). The antioxidant capacity is a complementary test that measures the ability of polyphenols to reduce a specific radical [30]. According to our results, the presence of azithromycin, hydroxychloroquine, ivermectin, and ciprofloxacin in the wine must reduced the antioxidant capacity by 75%, 50%, 46%, and 42% compared with the control (wine must without antibiotics), respectively. Similar to the behaviour of polyphenols, the presence of antibiotics affects the normal metabolism of yeasts; consequently, the antioxidant capacity decreases.

### 2.2. Effect of the Concentration of Antibiotics on the Content of Monomeric Anthocyanins

**Figure 3:**
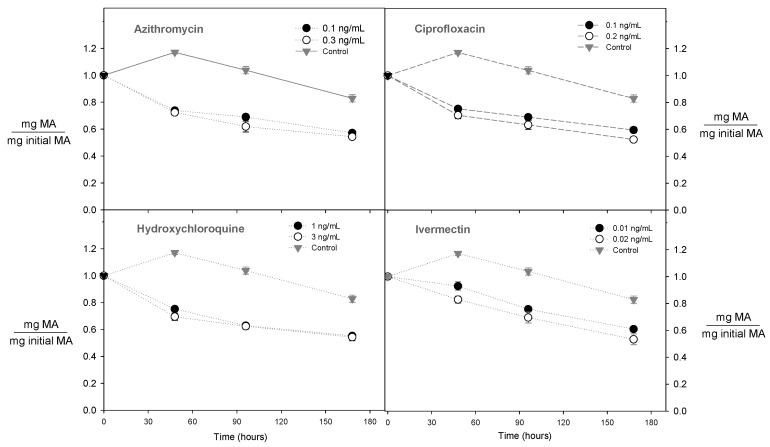
Effect of antibiotic type on total monomeric anthocyanins during the fermentation process.

Similar to the behaviour of the total polyphenols, the presence of azithromycin, hydroxychloroquine, ivermectin, and ciprofloxacin in the wine must reduced the content of monomeric anthocyanins by 38, 40, 31, and 41% compared with the control in the wine must, respectively (Figure 3). These results were likely associated with two factors, the conversion of monomeric anthocyanins into low molecular weight compounds and the effect of the antibiotics on the release of these compounds. In this sense, when the wine must without antibiotics (control) was evaluated during the natural fermentation process, the anthocyanin content decreased by ~15% (Figure 3). This behaviour could be explained because the monomeric anthocyanins may have been further converted into pyranoanthocyanins and other compounds with a low molecular weight such as acetylated, coumaroylated, and caffeoylated anthocyanins. Pyranoanthocyanins are the most abundant during the fermentation process and they can change the wine colour from bright red to brick and dark red [31]. Thus, the wine must without antibiotics presented a small decrease in the monomeric anthocyanins. On the other hand, the presence of antibiotics affected the metabolic ability of the yeasts to produce ethanol and CO_2_; consequently, a higher decrease in the anthocyanin content was observed.

### 2.3. Diffusion Kinetics of Polyphenols during the Fermentation Process

**Figure 4:**
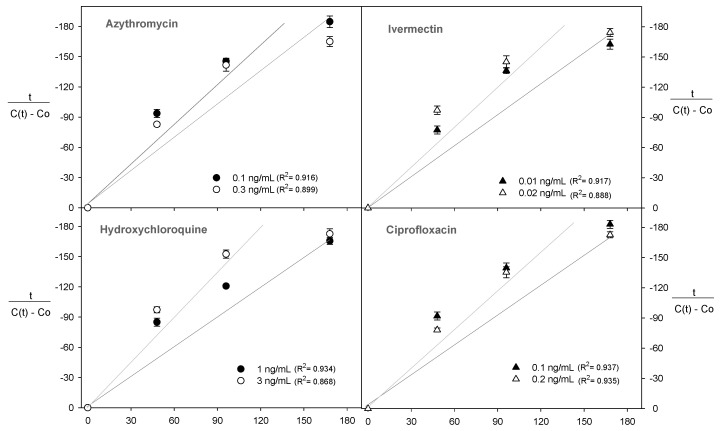
Peleg’s model linearisation of the total polyphenol content, where t corresponds with the time (min), C(t) is the concentration of polyphenols at time t, and C_0_ is the concentration of polyphenols at zero time.

The polyphenol content was evaluated over 168 h during the fermentation process and was linearised using the Peleg equation (Figure 4). This allowed us to determine the kinetic parameters k_1_ and k_2_ (R^2^ > 0.85) to explain the effect of the presence of antibiotics on the diffusion kinetics of the polyphenols (Figure 4).

For all the conditions studied, the Peleg rate constant (k_1_) increased with the antibiotics in the wine during fermentation (Table 1). The use of 3 ng/mL hydroxychloroquine was 6, 18, and 22% more effective in increasing the k_1_ constant compared with the use of ivermectin (0.02 ng/mL), azithromycin (0.3 ng/mL), and ciprofloxacin (0.2 ng/mL), respectively (Table 1). In this case, the kinetics of the analyte transfer were inversely proportional to the constant k_1_ [32]. Therefore, a higher value of k_1_ indicated a lower transfer process of polyphenols to the wine must.

Although the action mechanisms of antibiotics on wine yeasts are still a matter of discussion, it is essential to establish that the presence of low doses of antibiotics in drinking water can affect the metabolic mechanism of yeasts during the fermentation process. This could affect the sensory quality and polyphenolic content of the wine, a product that is in high demand for its bioactive properties.

### 2.4. Diffusion Kinetics of the Monomeric Anthocyanins

**Figure 5:**
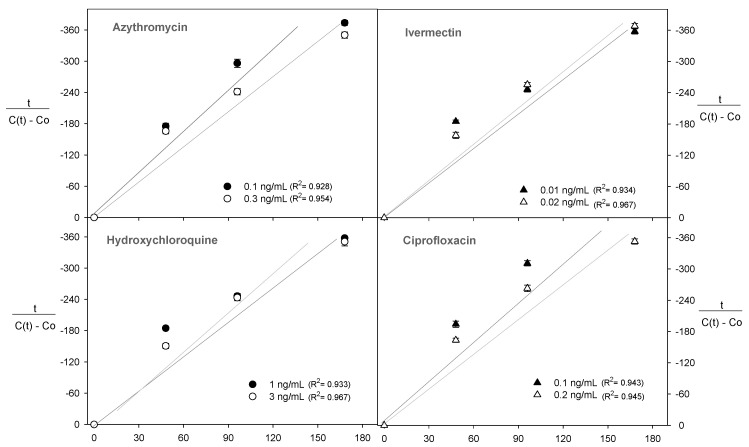
Peleg’s model linearisation of the total monomeric anthocyanin content, where t corresponds with the time (min), C(t) is the concentration of anthocyanin at time t, and C_0_ is the concentration of anthocyanin at zero time.

Although the presence of antibiotics in the wine must for a period of 168 h reduced the release of monomeric anthocyanins, it is necessary to explain this behaviour through the kinetic parameters of the Peleg model, such as k_1_ and k_2_, where the correlations were high in all experiments (R^2^ > 0.90) (Figure 5).

For all experiments, an increase in the antibiotic concentration increased the values of k_1_ and k_2_ (Table 2 and Figure 5). These results showed the influence of antibiotics on the extraction rate of monomeric anthocyanins into the wine must. The Peleg rate constant (k_1_) increased with the presence of antibiotics in the wine must during the fermentation process (Table 2). The use of ivermectin (0.02 ng/mL) increased by ~85% the value of the k_1_ constant compared with the use of hydroxychloroquine (3 ng/mL), azithromycin (0.3 ng/mL), and ciprofloxacin (0.2 ng/mL), respectively (Table 2). The antibiotics hydroxychloroquine, azithromycin, and ciprofloxacin specifically target bacteria whereas ivermectin is an antimicrobial that encompasses a broader range of organisms such as bacteria, yeast, fungi, and viruses [33,34]. Thus, ivermectin had a more remarkable ability to inhibit the yeast metabolism.

On the other hand, k_2_ as Peleg’s capacity constant was related to the maximum diffusion of anthocyanins during the fermentation process. In this sense, independent of the antibiotic concentration, the maximum diffusion was established between 0.92 and 1.02 (Table 2).

### 2.5. Impact of the Presence of Antibiotics on the Alcoholic Degrees in Wine

**Figure 6:**
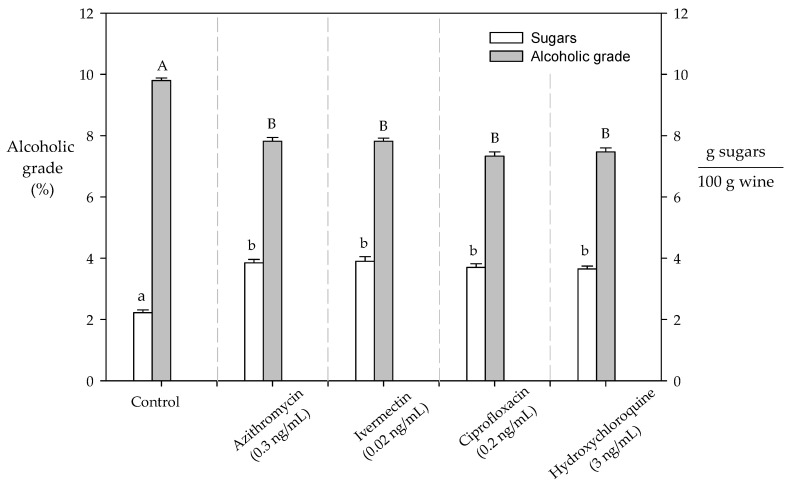
Alcoholic degrees at the end of the fermentation process. The significant differences (*p* < 0.05) are established with different lower-case letters and capital letters for the sugar content and alcoholic grade, respectively.

The wine alcohol concentration is related to the sugar concentration present in wine must because glucose and fructose are converted into ethanol through the metabolic pathways [35]. According to our results, the presence of antibiotics in the wine must affected the production of ethanol by ~26% during the fermentation process (Figure 6). In general, 1 mol of hexose sugar is converted into 2 mol ethanol and 2 mol CO_2_, which allowed us to establish that 0.51 g ethanol was produced per g of hexose sugar [36]. However, the presence of contaminants can affect ethanol formation due to incomplete fermentation and the accumulation of toxic compounds [36,37]. In this sense, it is probable that the presence of antibiotics inhibits the yeast metabolism, thus reducing the ethanol production.

## 3. Methodology

### 3.1. Sample

Twenty kilograms of grapes (*Vitis vinifera* L. cv. Negra Criolla) were purchased from the local market in the cities of Ilo and Moquegua in Peru. The grapes were selected, washed, and stored at 5 °C before making the wine.

### 3.2. Reactives

2,2′-azino-bis (3-ethylbenzothiazoline-6-sulphonic acid) (ABTS) and 2 N Folin–Ciocalteu were purchased from Sigma Chemicals (St. Louis, MO, USA). The standards were azithromycin, ivermectin, hydroxychloroquine, ciprofloxacin, and gallic acid, purchased from Sigma Chemicals (St. Louis, MO, USA). Potassium chloride was purchased from Supelco. Sodium acetate trihydrate was obtained from DUKSAN Pure Chemicals (Sungkok-Dong, South Korea). Sodium hydroxide was purchased from Sigma Chemicals. All solvents and other chemicals were purchased from Merck (Darmstadt, Germany) and J.T. Baker (Trinidad and Tobago). Ultra-purified water (resistivity of 18.2 MΩ.cm and total organic carbon < 10 ppb) was also used.

### 3.3. Preparation of Wine with the Addition of Antibiotics in Drinking Water

Low-alcohol wines were produced according to the methodology proposed by Schelezki et al. [19]. The grapes were washed, crushed, and macerated for 12 h. The grape must was then transferred and corrected by adding ultra-purified drinking water (Water Purification System, Dionex™ IC, Thermo Scientific, San Jose, CA, USA), which contained different doses of antibiotics such as azithromycin (0.1 and 0.3 ng/mL), hydroxychloroquine (1.0 and 3.0 ng/mL), ivermectin (0.01 and 0.02 ng/mL), and ciprofloxacin (0.1 and 0.2 ng/mL) (Table 3).

The correction was made using 68% wine must and 32% ultra-purified drinking water. After 0.3 g/L of yeast (*Sacharomyces ceresevisiae* var *cerevisiae*) was inoculated into the wine must, the fermentation process was started at 24 °C for 168 h. The samples were taken at different periods (0, 48, 96, and 168 h) (Figure 1). Finally, the samples were protected from light and stored at −20 °C until further analysis.

### 3.4. Total Polyphenol Content

The polyphenol analysis was performed using the methodology proposed by Singleton and Rossi [39]. A total of 500 µL of the sample, 250 µL of the Folin–Ciocalteu reactive (1 N), and 1250 µL of sodium carbonate (1 N) were mixed. The sample was then protected from light for 30 min at room temperature. After this, the samples were analysed at 765 nm in a UV-visible spectrophotometer. The results were expressed as mg of equivalent gallic acid per mL of wine. Simultaneously, a standard curve was obtained using gallic acid as a reference standard.

### 3.5. Monomeric Anthocyanin Content

The differential pH method was used to determine the monomeric anthocyanin content, according to Da Fonseca Machado et al. [40] with a few modifications. The samples were centrifuged at 4000× *g* rpm for 10 min and were diluted in two buffer solutions, potassium chloride pH 1.0 (0.025 mol/L) and sodium acetate pH 4.5 (0.40 M); both were adjusted with concentrated hydrochloric acid. The absorbance was then measured at 520 and 700 nm. The quantification of the monomeric anthocyanin content was calculated using the following Equation (1) and the results were expressed as mg cyanidin-3-glucoside equivalent/L.

(1) $$\mathrm{mg}\;\mathrm{cyanidin}-3-\mathrm{glucoside}\;\mathrm{equivalent}/L=\frac{A\;\times MW\;\times DF}{\epsilon \;\times L}$$

where *A* is (Abs510 _ Abs700) pH 1.0 − (Abs510 _ Abs700) pH 4.5; *MW* is 449.2 g/mol (molar mass of cyanidin-3-O-glucoside); *DF* is the dilution factor determined for the buffer solutions; ϵ is 26,900 L/cm·mol (molar absorptivity of cyanidin-3-Oglucoside); and *L* is the correction factor for a 1 cm optic path length.

### 3.6. Determination of Antioxidant Capacity (ABTS)

The antioxidant capacity was evaluated using the ABTS + 2 methodology proposed by Arnao et al. [41]. A total of 150 µL of the sample was mixed with 2850 µL of the ABTS solution. The absorbance of each of the samples was then measured at 734 nm. The antioxidant capacity was determined using a Trolox standard curve. The results were expressed as µmol Trolox equivalent per mL of wine must.

### 3.7. Determination of Alcoholic Degrees and Sugar Content

The samples were analysed to determine the alcoholic degrees and sugar content according to methods referred to in the technical standards NTP 212.030: 2009 and NTP 212.038: 2009 [42].

### 3.8. Evaluation of the Diffusion Kinetics of Polyphenols in the Wine Must

The diffusion kinetics of the total polyphenols in the wine must during the fermentation process were analysed using the Equation (2) proposed by Peleg [24]:

(2) $$\frac{t}{C_{\left(t\right)}-C_{0}}=k_{1}+k_{2}t$$

where t is the time in minutes, C_(t)_ is the concentration of polyphenols (mg/mg_0_) at time t, C_0_ is the initial polyphenol concentration, k_1_ is the Peleg kinetic rate constant, and k_2_ is the Peleg capacity constant. A linear regression was performed to validate the adjustment of the model considering an R^2^ > 0.85.

### 3.9. Statistical Analysis

A completely random design was applied to determine the behaviour of the antibiotic addition on the content of the antioxidant compounds. The results were presented as a mean and standard deviation. A one-way ANOVA and a Tukey comparison test were then applied to establish the significant differences (*p* < 0.05). The statistical analysis was performed using the Statgraphics Plus for Windows 4.0 program (Stat Point Technologies, Inc., Warrenton, VA, USA).

## 4. Conclusions

The presence of antibiotics in drinking water significantly reduced the diffusion of the total polyphenols and monomeric anthocyanins into the wine must between 40 and 80%, respectively. The Peleg kinetic parameters allowed the linearisation of the diffusion of polyphenols and anthocyanins. This confirmed that the use of antibiotics reduced the diffusion of these compounds in the wine must. Interestingly, ivermectin was more effective in reducing the diffusion of anthocyanins into the wine must. It is probable that the chemical structure and molecular weight played a decisive role in the inhibition of the yeast metabolism. Thus, it is necessary to establish strategies to avoid the presence of antibiotics in the treatment of drinking water to not affect its use in wine processing. Finally, our results contribute to the simulation and optimisation of the fermentation process.

## Figures and Tables

**Table 1:** Peleg’s constants of the diffusion of polyphenols during the fermentation process.

Description		k_1_ (h.mg/mg_0_) Mean CV	k_2_ (mg/mg_0_) Mean CV
Azithromycin	0.1 ng/mL	21.76 ^c^ 0.01	1.07 ^b^ 0.01
	0.3 ng/mL	22.84 ^c^ 0.01	0.97 ^a,b^ 0.01
Ivermectin	0.01 ng/mL	19.32 ^b^ 0.02	0.95 ^a^ 0.03
	0.02 ng/mL	26.22 ^d^ 0.01	0.99 ^a,b^ 0.02
Hydroxychloroquine	1 ng/mL	19.27 ^b^ 0.02	0.94 ^a^ 0.03
	3 ng/mL	27.83 ^e^ 0.02	0.99 ^a,b^ 0.00
Ciprofloxacin	0.1 ng/mL	17.39 ^a^ 0.03	0.92 ^a^ 0.02
	0.2 ng/mL	21.47 ^c^ ± 0.03	0.93 ^a^ ± 0.03

The results are expressed as the mean and CV (coefficient of variation). Different lower-case letters indicate significant differences (*p* < 0.05) between treatments.

**Table 2:** Peleg’s constants of the diffusion of monomeric anthocyanin content during the fermentation process.

Description		k_1_ (h.mg/mg_0_) Mean CV	k_2_ (mg/mg_0_) Mean CV
Azithromycin	0.1 ng/mL	32.95 ^b^ 0.01	2.00 ^c^ 0.01
	0.3 ng/mL	40.58 ^c^ 0.01	2.19 ^d^ 0.00
Ivermectin	0.01 ng/mL	90.12 ^d^ 0.02	0.95 ^a^ 0.03
	0.02 ng/mL	278.32 ^e^ 0.00	1.51 ^b^ 0.01
Hydroxychloroquine	1 ng/mL	28.45 ^a^ 0.02	1.93 ^c^ 0.02
	3 ng/mL	39.79 ^c^ 0.01	2.14 ^d^ 0.00
Ciprofloxacin	0.1 ng/mL	34.81 ^b^ 0.01	2.05 ^c^ 0.01
	0.2 ng/mL	41.95 ^c^ 0.01	2.41 ^e^ 0.01

The results are expressed as the mean and CV (coefficient of variation). Different lower-case letters indicate significant differences (*p* < 0.05) between treatments.

**Table 3:** Selection of levels according to other research works.

Antibiotics	Levels Used in This Study (ng/mL)	Levels Reported (ng/mL)	References
Ciprofloxacin	0.1 and 0.2	0.02–0.22	Boleda et al. [8]
Azithromycin	0.1 and 0.3	0.17–0.57	Boleda et al. [8]
Ivermectin	0.01 and 0.02	0.005–0.02	Nippes et al. [9]
Hydroxychloroquine	1.0 and 3.0	0.78–3.00	Morales et al. [38]

## Data Availability

Not applicable.
